# The Burden of Sarcoidosis Symptoms from a Patient Perspective

**DOI:** 10.1007/s00408-019-00206-7

**Published:** 2019-02-16

**Authors:** M. Voortman, C. M. R. Hendriks, M. D. P. Elfferich, F. Bonella, J. Møller, J. De Vries, U. Costabel, M. Drent

**Affiliations:** 1 ILD Center of Excellence, Department of Pulmonology, St. Antonius Hospital, PO Box 2400, 3430 VB Nieuwegein, The Netherlands; 2 Department of Pulmonology, Division of Heart & Lungs, University Medical Centre Utrecht, Utrecht, The Netherlands; 3 ILD Care Foundation Research Team, Ede, The Netherlands; 4 Faculty of Medicine, Utrecht University, Utrecht, The Netherlands; 5 Interstitial and Rare Lung Disease Unit, Ruhrlandklinik, University Hospital, Essen, Germany; 6 Department of Respiratory Diseases and Allergology, Aarhus University Hospital, Aarhus, Denmark; 7 Department of Medical Psychology, Elisabeth-TweeSteden Hospital Tilburg, Tilburg, The Netherlands; 8 Department of Medical and Clinical Psychology, Tilburg University, Tilburg, The Netherlands; 9 Department of Pharmacology and Toxicology, FHML, Maastricht University, Maastricht, The Netherlands

**Keywords:** Fatigue, Fatigue Assessment Scale (FAS), Sarcoidosis, Sarcoidosis-associated symptoms, Small fiber neuropathy (SFN), Small fiber neuropathy screenings list (SFNSL)

## Abstract

**Purpose:**

The clinical manifestations of sarcoidosis vary widely, depending on the intensity of the inflammation and the organ systems affected. Hence, sarcoidosis patients may suffer from a great variety of symptoms. The aim of this study was to compare the self-reported burden of sarcoidosis patients in Denmark, Germany and the Netherlands, especially the prevalence of fatigue and small fiber neuropathy (SFN)-related symptoms, as well as differences in treatment strategies.

**Methods:**

A cross-sectional web-based anonymous survey about complaints was conducted among sarcoidosis patients. Patients were invited to take part through the sarcoidosis patient societies as well as through outpatient sarcoidosis clinics in these countries.

**Results:**

The questionnaire was completed by 1072 sarcoidosis patients (152 Danish, 532 German and 388 Dutch). Almost all patients reported having sarcoidosis-associated symptoms (organ-related as well as non-specific, non-organ related). Fatigue was reported by almost all respondents (90%), followed by pulmonary symptoms (72.4%). More than 50% of the respondents were being treated with prednisone, which was comparable in all three countries. In contrast, second- and third-line treatment differed substantially between Denmark, Germany and the Netherlands.

**Conclusion:**

Sarcoidosis patients in Denmark, Germany and the Netherlands present with similar self-reported symptoms, organ-related as well as non-specific, non-organ related. Fatigue (90%) and symptoms associated with SFN (86%) were highly prevalent in all three countries.

**Electronic supplementary material:**

The online version of this article (10.1007/s00408-019-00206-7) contains supplementary material, which is available to authorized users.

## Introduction

Sarcoidosis occurs throughout the world, affecting all races and ages. Its true prevalence remains unknown. To date, studies based on nationwide registries of demographic factors and diagnoses are relatively old and scarce [1–4]. Moreover, the epidemiological assessment of sarcoidosis and its manifestations is problematic due to lack of consistent case definition, lack of sensitivity and specificity of diagnostic tests, variable diagnostic intensity and variable diagnostic methods [5].

The clinical manifestation, natural history and prognosis of sarcoidosis are highly variable, and its course is often unpredictable [6]. Depending on the organs involved and the severity of granulomatous inflammation, patients suffer from a broad range of symptoms. In addition to organ-related symptoms, patients often suffer from disabling non-specific, non-organ-related symptoms [7–9]. Fatigue is the most frequently reported non-specific burdensome symptom in sarcoidosis patients, with a prevalence ranging from 50 to 90% [10, 11]. Other non-specific symptoms accounting for an important impact on the quality of life (QoL) of both patients and partners are small fiber neuropathy (SFN)-related symptoms and everyday cognitive failure, including concentration problems, memory loss and decreased perception [7, 12–15]. Moreover, clinicians tend to have more attention for physical parameters than psychological issues in patients with sarcoidosis. To date, drugs used to treat severe organ involvement in sarcoidosis generally do not influence these non-specific symptoms and tend to cause side effects which then further increase the burden of disease.

The importance of patients’ participation in healthcare has been increasingly acknowledged. Moreover, studies from a patients’ perspective are important. Patients reported they regularly feel misunderstood and would like more attention and support for their problems [13]. Therefore, the aim of this study was to assess the self-reported burden of patients with sarcoidosis in three European countries, viz. Denmark, Germany and the Netherlands, especially regarding the prevalence of fatigue and SFN-related symptoms, as well as differences in treatment strategies.

## Materials and Methods

### Study Design

In cooperation with the Dutch Sarcoidosis Society, the ild care foundation has designed a questionnaire to assess complaints (organ-related as well as non-specific, non-organ related) among patients with sarcoidosis. This cross-sectional web-based anonymous survey was conducted from October 2017 to April 2018 among a sample of sarcoidosis patients in the Netherlands, and from December 2017 to August 2018 among samples of sarcoidosis patients in Denmark and Germany. The recruitment procedure aimed to compose representative samples of sarcoidosis patients in these countries.

This study was performed in accordance with the Declaration of Helsinki and its amendments. The Medical Ethics Committee of the St. Antonius Hospital Nieuwegein, the Netherlands, decided that, under the Dutch act on medical research involving human subjects, approval of this study by a Medical Ethics Committee was not necessary.

### Study Subjects and Procedure

The overall study sample comprised sarcoidosis patients who were members of the Dutch Sarcoidosis Society (total number of patient members about 2000) and the Deutsche Sarkoidose Vereinigung (total number of patients members about 4000), and mainly from a sarcoidosis clinic in Denmark. Patients were recruited without incentives, since the survey was anonymous.

The survey was developed using the online questionnaire tool *Surveymonkey* (http://www.surveymonkey.com). The survey concerns the burden of disease and symptoms as experienced by patients with sarcoidosis. Further questions concerned demographics (gender, age, duration of sarcoidosis), use of medication and two sets of questionnaires validated for sarcoidosis, the Small Fiber Neuropathy Screening List (SFNSL) [16] and the Fatigue Assessment Scale (FAS) [17]. Patients were provided with a link to the electronic survey.

### Questionnaires

The FAS is a 10-item self-report fatigue questionnaire. The response scale is a five-point scale (1 never to 5 always); scores on the FAS can range from 10 to 50. A score > 22 indicates fatigue, and a score > 34 indicates extreme fatigue. The reliability and validity of the FAS have been shown to be good in sarcoidosis patients [17]. So far, the FAS is available in 20 languages [18] (see Supplement 1and http://www.wasog.org/education-research/questionnaires.html).

The SFNSL is a 21-item self-administered questionnaire to screen for symptoms related to SFN. The response scale is a five-point scale (0 never to 4 always); scores on the SFNSL can range from 0 to 84. The cutoff score of the SFNSL is 11: a score below 11 indicates no or few symptoms related to SFN, while a score of 11–48 indicates probable or highly probable SFN and a score above 48 is indicative of SFN [16]. The SFNSL is available in six languages: Danish, Dutch, English, French, German and Japanese (see Supplement 1 and http://www.wasog.org/education-research/questionnaires.html).

### Statistical Analysis

All statistical analyses were performed using SPSS version 24 for Mac. Standard descriptive statistics were computed. ANOVA was used for comparison between the sarcoidosis samples in the three countries. In view of the large number of variables examined, a probability value of less than 0.01 was considered to be statistically significant.

## Results

The characteristics of the samples studied are summarized in Table 1. A total of 1072 sarcoidosis patients [152 (68% female) Danish; 532 (62% female) German and 388 (53% female) Dutch] completed the survey. The mean age was 51.8 years and did not differ between the three countries.

**Table 1:** Summary of the characteristics of the sarcoidosis patient samples from Denmark, Germany and the Netherlands

	Denmark	Germany	The Netherlands	p value^#^
Number	152	532	388	
Gender, male (%)	31.9	38.0	46.6	< 0.001
Age, years, mean ± SD (range)	52.1 ± 9.9 (12–72)	52.3 ± 9.2 (23–80)	51.1 ± 9.7 (24–75)	NS
Organ involvement (%)				
Pulmonary	75.0	73.0	69.1	NS
Cardiac	2.1	7.8	5.4	< 0.001
Musculoskeletal	80.9	69.3	60.5	< 0.001
Skin	31.6	35.8	27.3	NS
Low vitamin D	27.9	31.7	20.4	0.002
Hypercalcemia	9.6	4.5	6.7	NS
Nervous system	10.3	11.9	11.6	NS
Liver	4.4	14.8	6.7	< 0.001
Symptoms (%)				
None	4.4	4.7	1.5	NS
Organ-related symptoms (%)	95.6	95.3	95.9	NS
Pulmonary				
Dyspnoea	65.4	62.3	61.1	NS
Cough	49.3	42.1	38.1	0.05
Extrapulmonary	95.6	94.6	90.7	NS
Cardiac arrhythmia*	14.7	18.7	28.4	< 0.001
Dizziness/fainting	33.8	29.4	29.3	< 0.001
Kidney stones	5.1	5.1	6.2	NS
Non-organ-related symptoms (%)	95.6	97.7	94.4	NS
Fatigue	89.9	89.7	90.7	NS
Pain	74.5	68.9	62.5	< 0.001
Reduced energy levels	80.9	82.1	78.7	NS
Concentration problems	51.5	54.2	56.2	NS
Memory problems	58.1	46.9	47.9	0.05
Sleeping problems	56.6	50.4	44.6	NS
Restless legs	36.8	32.1	33.2	NS
Dry or running eyes	43.4	42.6	39.7	NS
Medication (%)				
None, ever	27.1	32.0	33.2	NS
Predniso(lo)n	56.6	51.4	54.8	NS
Methotrexate	23.5	8.5	21.7	< 0.001
Azathioprine	7.4	9.2	3.8	0.01
Hydroxychloroquine	5.9	0.1	4.9	< 0.001
Anti-TNF-alfa	4.0	2.6	7.2	< 0.001
Vitamin D	39.1	40.5	29.8	< 0.001
Calcium	38.0	10.5	10.0	NS (DN vs. GE and DN vs. NL < 0.001)
Pain killers or nsaids	35.3	48.6	32.7	0.005
Sleeping medication	5.9	4.3	8.5	NS

*TNF* tumor necrosis factor, *NS* nonsignificant, *NSAID* non-steroidal anti-inflammatory drug; *DN* Denmark, *GE* Germany, *NL* the Netherlands

*Patient’s experience not confirmed by a cardiologist

^#^Significantly different between all three countries (the Netherlands and Denmark and Germany)

Self-reported pulmonary involvement was the most common complaint (72.4%), followed by musculoskeletal involvement (70.2%). Rates of musculoskeletal, cardiac and liver sarcoidosis differed between the three European countries.

Almost all patients (95%) reported having symptoms (organ-related as well as non-specific, non-organ related). Of the non-specific, non-organ-related symptoms, fatigue was reported by almost all sarcoidosis patients in all three countries (approximately 90%). SFN-related symptoms (86.2%), reduced energy levels (80.6%) and concentration (54.0%), memory (51%) and sleeping (50.5%) problems were also often reported. None of these symptoms varied between the three European countries. Only memory problems tended to be more prevalent in Denmark (58.1%, *p* = 0.05). Significant differences were found regarding self-reported pain, which was least prevalent in the Netherlands (*p* ≤ 0.001), and low vitamin D levels, with the highest prevalence in Germany (31.7%, *p* = 0.002).

More than 50% of the studied sample were being treated with prednisone, a rate which was comparable in all three countries. However, substantial differences were found concerning second- and third-line treatment. Methotrexate (MTX) was used more commonly in Denmark and the Netherlands than in Germany [23.5% (DN) and 21.7% (NL) vs. 8.5%; *p* ≤ 0.001], while azathioprine (AZA) was prescribed more often in Germany [9.2% vs. 7.4% (DN) and 3.8% (NL); *p* ≤ 0.001]. TNF-alpha inhibitors were used most frequently in the Netherlands [7.2% vs. 4.0% (DN) and 2.6% (GE); *p* ≤ 0.001]. In total, 34.9% of the patients in Denmark, 32.7% of the patients in the Netherlands and 20.3% of the patients in Germany were receiving second- or third-line treatments. The use of calcium supplementation was higher in Denmark [38.0% (DN) vs. 10.5% (GE) and 10.0% (NL); *p* ≤ 0.001], and more German patients reported using pain killers (including opioids).

**Fig. 1:**
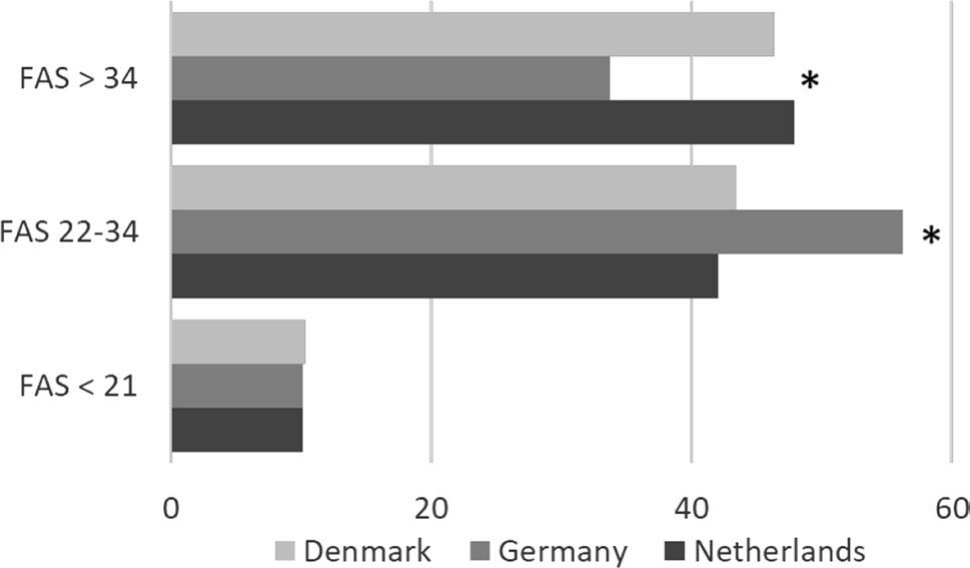
Fatigue Assessment Scale (FAS) subscores per country. FAS scores below 22 indicate no fatigue, scores between 22 and 34 indicate mild to moderate fatigue, and scores above 34 indicate severe fatigue [18]. * = p value < 0.001 (GE vs. DN + NL)

Based on the assumption that an FAS score above 22 indicates fatigue, approximately 90% of the sarcoidosis patients in all three countries were affected by fatigue. The mean FAS score in the three European countries was 32.1 (the highest score being found in the Netherlands: 33.1; *p* ≤ 0.001; see Table 2). Patients in the Netherlands and Denmark reported more extreme fatigue (FAS > 34) than those in Germany [47.9% (NL) and 46.3% (DN) vs. 33.7% (GE); *p* ≤ 0.001] (see also Fig. 1).

**Table 2:** Fatigue Assessment Scale (FAS) and small fiber neuropathy screening list (SFNSL) scores of the sarcoidosis samples from Denmark, Germany and the Netherlands

	Denmark	Germany	The Netherlands	p value
FAS, total ± SD	32.3 ± 8.4	30.8 ± 7.7	33.1 ± 8.1	< 0.001
FAS, mental ± SD	14.6 ± 4.6	13.8 ± 4.2	15.0 ± 4.4	< 0.001
FAS, physical ± SD	17.7 ± 4.5	17.4 ± 9.1	18.1 ± 4.2	NS
SFNSL, total ± SD	32.4 ± 16.4	26.6 ± 17.8	27.9 ± 18.7	0.004

*SD* standard deviation, *NS* nonsignificant

**Fig. 2:**
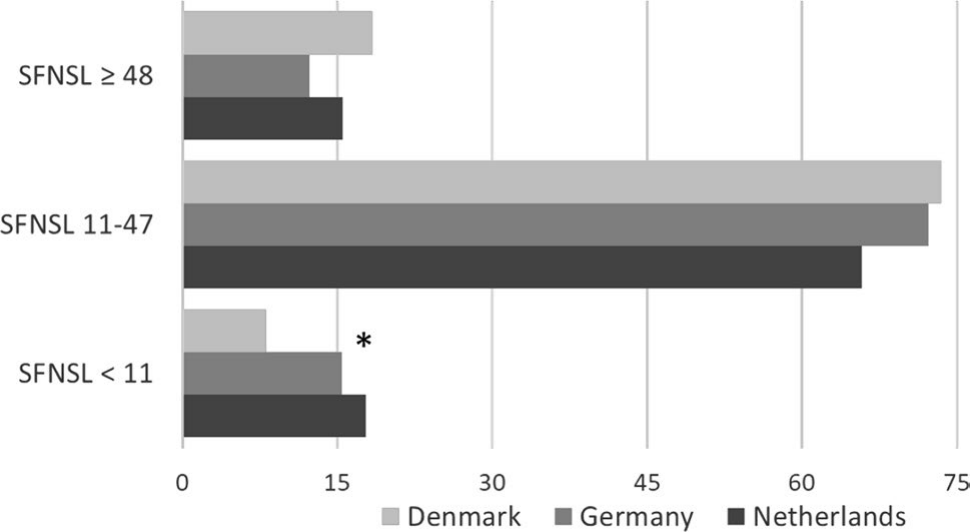
Small Fiber Neuropathy Screening List (SFNSL) subscores per country. A score below 11 indicates few or no SFN-related symptoms, a score between 11 and 48 indicates probable or highly likely SFN, and a score above 48 is indicative of SFN [16] * = p value < 0.001 (DN vs. GE + NL)

Based on the assumption that an SFNSL score > 11 indicates probable or highly probable SFN, > 80% of the sarcoidosis patients in all three countries are affected by SFN-related symptoms. The mean SFNSL score in the three European countries was 29 (the highest score being found in Denmark: 32.4; *p* ≤ 0.004). The highest prevalence of SFN-related symptoms was seen in Denmark (91.9%), followed by Germany (84.6%). In the Netherlands, 82.2% of the patients reported SFN-related symptoms (see also Table 2). The prevalence of scores > 48 (indicative of SFN) was also substantial: 18.5% in Denmark, 12.3% in Germany and 15.5% in the Netherlands (see also Fig. 2).

## Discussion

Our findings demonstrate that the self-reported burden of sarcoidosis is high. Almost all patients reported sarcoidosis-associated symptoms (organ-related as well as non-specific, non-organ related). Of the non-specific symptoms, fatigue (assessed by the FAS) and reduced energy levels were reported by almost all sarcoidosis patients in the three European countries we studied (approximately 90 and 80%, respectively). In addition, over 80% of the respondents reported SFN-related symptoms (as assessed by the SFNSL). Pulmonary involvement was the most frequently organ-related manifestation reported, followed by musculoskeletal manifestations.

Difficulties arise when estimating the real extent and prevalence of organ involvement and non-specific symptoms associated with sarcoidosis in a community. Descriptions differ widely among populations in the world and even between various regions within countries, as standardization of the diagnostic criteria is still lacking [1]. To date, epidemiological data on the prevalence of various manifestations in the European countries we studied are sparse. In Denmark, 50% of the cases in a National Patient Registry were apparently asymptomatic [1]. New imaging tests such as PET scans, revealing occult sarcoidosis localizations and/or multiple organ involvement, have led to changes in reported manifestations [5, 19]. In the present study, the reported proportion of organ involvement was comparable between the three countries, except for cardiac and liver involvement (both most prevalent in Germany).

Clinically apparent cardiac involvement has been noted in 2–10% of patients [20], while the prevalence of clinically silent cardiac sarcoidosis is much higher. Advanced imaging, including PET scans and magnetic resonance imaging (MRI), has improved the detection of cardiac sarcoidosis [20]. Apparently, there are differences in the presentation of sarcoidosis in this respect between patients from Europe and America and those from Japan, as Japanese studies have reported much higher rates of cardiac involvement. The prevalence of cardiac involvement reported in the present study was similar to previously reported rates (8%) in large sarcoidosis samples in Germany [2, 21]. In contrast, the present study showed a rather lower prevalence in Denmark (2.1%), which is not consistent with previously reported rates in Denmark (8.6%) [22]. This difference may be due to the fact that our Danish study sample as well as the previous study was conducted in a single center [22]. Moreover, the prevalence of manifestations also depends on the available diagnostic procedures, the expertise of the clinicians and the definition used to determine organ involvement in various centers.

Liver problems in sarcoidosis could be associated with, at least partly, hepatic sarcoidosis, hepatotoxic medication such as azathioprine and/or alcohol intake. In line with the findings of the present study, Kirsten and Bosse-Henck et al. reported high rates of liver involvement (15%) in large sarcoidosis samples in Germany [2, 21]. Recently, liver involvement was found in approximately 15% of a Dutch sarcoidosis sample, which is higher than reported in the present study (6.7%) [23]. This can probably be explained by the fact that the sample studied by Cremers et al. included more chronic, advanced sarcoidosis patients, since their study was performed in a tertiary referral center. So far, comparative epidemiologic data on liver involvement in Denmark are lacking.

Organ-specific symptoms were comparable between the three countries, except for musculoskeletal symptoms (being most prevalent in Denmark). Musculoskeletal manifestations occur in approximately one-third of sarcoidosis patients [24]. Since they are often subclinical or not recognized because of mild or non-specific symptoms, however, their exact prevalence is unknown. The most frequent musculoskeletal manifestation of sarcoidosis is an acute arthritis that occurs as part of the Löfgren syndrome [24]. Higher rates of musculoskeletal involvement have been reported since the introduction of the PET-CT [25]. Awareness of organ involvement and sarcoidosis-associated symptoms will continue to increase with the improvement in diagnostic options and more prominent patient participation in the management of their disease.

In line with previous studies, the present study found that non-specific symptoms were frequently reported by sarcoidosis patients [9–11, 13, 14, 26, 27]. Hinz et al. reported fatigue in 70% of the German sarcoidosis population (mean FAS score: 26.3 [26]) and De Kleijn et al. reported fatigue in 83% of the Dutch sarcoidosis population (mean FAS score: 30.3 [28]). To date, ours was the first study evaluating fatigue in Denmark, so no previous data exist. These findings underline the clinical importance of assessing fatigue in sarcoidosis and integrating it in the multidisciplinary management of this disease. The etiology of sarcoidosis-associated fatigue is poorly understood and is likely to be multifactorial, encompassing active inflammation, cytokine release, depressive symptoms, sleep disturbance, anxiety, everyday cognitive failure and/or SFN-related symptoms [7, 11]. Fatigue can also be caused by systemic treatments for sarcoidosis, such as corticosteroids [29, 30]. Recently, our group demonstrated that predictors of fatigue include everyday cognitive failure, SFN-associated symptoms, depressive symptoms, anxiety, muscle pain and dyspnea [8]. Although our survey did not include a depressive symptoms inventory, it can be concluded from earlier studies by our group and others that the presence of depressive symptoms is one of the most important predictors of fatigue [8, 21]. Moreover, more than fifty percent of the patients in our sample reported memory and concentration problems, symptoms associated with cognitive failure and fatigue [8].

Ours was also the first study to assess SFN-related symptoms among sarcoidosis patients in Denmark and Germany. SFN-related symptoms, including pain, were frequently reported by all participants in the present study. In the follow-up of sarcoidosis patients, routine tests to assess disease activity do not measure pain, so the results of tests do not always correlate with the patients’ well-being [4]. Since pain is a substantial problem in sarcoidosis, appropriate questionnaires to obtain information about pain might be helpful [9].

The percentage of patients using drugs was comparable in the three European countries (around 70%). However, treatment strategies differed between these three countries, a difference which was largely caused by the difference in second- and third-line treatment [20% (GE) vs. 33% (NL) and 35% (DN)]. Patients in Denmark and the Netherlands were more likely to use methotrexate and TNF-alpha inhibitors, whereas in Germany azathioprine was regarded as the treatment of first choice when prednisone alone was not effective. Remarkably, TNF-alpha inhibitors were prescribed most frequently in the Netherlands. This can most probably be explained by differences between the three countries in the health care insurance systems and/or local guidelines.

## Limitations

One of the limitations of the present study was the patient recruitment method used in the three countries. The majority of the patients were recruited through patient societies. One could argue that it is only symptomatic patients who become members of a patients’ society or are specifically referred to a specialized sarcoidosis clinic, and so these were more likely to take part in the survey than asymptomatic patients, causing selection bias and considerably influencing the rate of symptomatic cases in this study. Therefore, these results are only applicable to symptomatic patients. At present, the rate of symptoms in the unselected nationwide sarcoidosis population remains unknown. Another limitation is the fact that the symptoms were self-reported, which could lead to bias. However, the burden of sarcoidosis is determined by the experience of the patients themselves, and we barely saw any differences in prevalence rates of sarcoidosis-associated symptoms between the European countries we studied.

## Conclusion

This study shows that sarcoidosis patients in Denmark (DN), Germany (GE) and the Netherlands (NL) report similar sarcoidosis-associated symptoms, including organ-related as well as non-specific, non-organ-related symptoms. Fatigue (90%) and symptoms associated with SFN (mean 86%: DN 91.9%, GE 84.6% and NL 82.2%, respectively) were highly prevalent in all three countries.

Beside better disease education, psychological support is warranted. The combined use of appropriate questionnaires, lung function tests, imaging procedures and other clinical assessments of disease activity and severity provides a framework for evaluating organ-related as well as non-organ-related symptoms.

In view of the broad range of possible symptoms, sarcoidosis patients may consult various doctors, so the management of sarcoidosis patients should use a multidisciplinary approach that focuses on somatic as well as psychosocial aspects of this erratic disorder. Furthermore, as treatment strategies differ in the three European countries we studied, updated international sarcoidosis treatment guidelines are urgently needed.

## Electronic supplementary material

Below is the link to the electronic supplementary material.

Supplementary material 1 (DOCX 23 KB)
