# Correction to: The Burden of Sarcoidosis Symptoms from a Patient Perspective

**DOI:** 10.1007/s00408-019-00221-8

**Published:** 2019-04-08

**Authors:** M. Voortman, C. M. R. Hendriks, M. D. P. Elfferich, F. Bonella, J. Møller, J. De Vries, U. Costabel, M. Drent

**Affiliations:** 1 ILD Center of Excellence, Department of Pulmonology, St. Antonius Hospital, PO Box 2400, 3430 VB Nieuwegein, The Netherlands; 2 Department of Pulmonology, Division of Heart & Lungs, University Medical Centre Utrecht, Utrecht, The Netherlands; 3 ILD Care Foundation Research Team, Ede, The Netherlands; 4 Faculty of Medicine, Utrecht University, Utrecht, The Netherlands; 5 Interstitial and Rare Lung Disease Unit, Ruhrlandklinik, University Hospital, Essen, Germany; 6 Department of Respiratory Diseases and Allergology, Aarhus University Hospital, Aarhus, Denmark; 7 Department of Medical Psychology, Elisabeth-TweeSteden Hospital Tilburg, Tilburg, The Netherlands; 8 Department of Medical and Clinical Psychology, Tilburg University, Tilburg, The Netherlands; 9 Department of Pharmacology and Toxicology, FHML, Maastricht University, Maastricht, The Netherlands

## Correction to: Lung 10.1007/s00408-019-00206-7

The original version of this article unfortunately contained a mistake in coauthor's given name. The abbreviated given name "M.D." for coauthor M.D.P. Elfferich's name has been inadvertently deleted during the production process.

The original article has been corrected.

