# Supplementary material for: The Burden of Sarcoidosis Symptoms from a Patient Perspective
Source: Lung. 2019 Feb 16;197(2):155–61. doi: 10.1007/s00408-019-00206-7 (PMC6486948; doi:10.1007/s00408-019-00206-7)
Supplement: Supplementary file 1 — Supplementary material 1 (DOCX 23 KB) [file 408_2019_206_MOESM1_ESM.docx]

**Supplement manuscript #LUNG-D-18-00439**

**Fatigue Assessment Scale**

The Fatigue Assessment Scale (FAS; Table 1) is a 10-item self-report fatigue questionnaire [1,2]. It is quick and easy to complete for patients, and not time consuming. An answer to every question has to be given, even if the person does not have any complaints at the moment. Five questions reflect physical fatigue and 5 questions (questions 3 and 6-9) mental fatigue. The response scale is a five-point scale (1 never to 5 always); scores on the FAS can range from 10 to 50. A score > 22 indicates fatigue and a score > 34 indicates extreme fatigue. Scores on question 4 and 10 should be recoded (1=5, 2=4, 3=3, 4=2, 5=1). Subsequently, the total FAS score can be calculated by summing the scores on all questions (recoded scores for questions 4 and 10). The reliability and validity of the FAS have been shown to be good in sarcoidosis patients [1]. So far, the FAS is available in 20 languages (see <http://www.wasog.org/education-research/questionnaires.html>) [3].

The Minimal Important Difference (MID) is at least 4 points or 10% change of the baseline value [4].

**Table 1. Fatigue Assessment Scale (FAS)**

The following ten statements refer to how you usually feel. Per statement you can choose one out of five answer categories, varying from Never to Always. Please circle the answer to each question that is applicable to you. Please give an answer to each question, even if you do not have any complaints at the moment.

**1. never**

**2. sometimes** (about monthly or less)

**3. regularly** (about a few times a month)

**4. often** (about weekly)

**5. always** (about every day)

|  | never | sometimes | regularly | often | always |
| --- | --- | --- | --- | --- | --- |
| \| 1. I am bothered by fatigue \| \| --- \| |  |  |  |  |  |
| \| 2. \| I get tired very quickly \| \| --- \| --- \| |  |  |  |  |  |
| \| 3. \| I don’t do much during the day \| \| --- \| --- \| |  |  |  |  |  |
| \| 4. \| I have enough energy for everyday life \| \| --- \| --- \| |  |  |  |  |  |
| \| 5. \| Physically, I feel exhausted \| \| --- \| --- \| |  |  |  |  |  |
| \| 6. \| I have problems to start things \| \| --- \| --- \| |  |  |  |  |  |
| \| 7. \| I have problems to think clearly \| \| --- \| --- \| |  |  |  |  |  |
| \| 8. \| I feel no desire to do anything \| \| --- \| --- \| |  |  |  |  |  |
| \| 9. \| Mentally, I feel exhausted \| \| --- \| --- \| |  |  |  |  |  |
| \| 10. \| When I am doing something, I can  concentrate quite well \| \| --- \| --- \| |  |  |  |  |  |

**Small Fiber Neuropathy Screenings List**

The small fiber neuropathy screenings list (SFNSL; table 2) was developed to assess symptoms which may be related to SFN, but not to diagnose SFN. The SFNSL is a 21-item self-administered questionnaire to screen for symptoms related to SFN. The response scale is a five-point scale (0 never to 4 always); scores on the SFNSL can range from 0 to 84. The cut-off score of the SFNSL is 11: a score below 11 indicates no or few symptoms related to SFN, while a score of 11-48 indicates probable or highly probable SFN and a score above 48 is indicative of SFN [5]. So far, the SFNSL is available in six languages; Danish, Dutch, English, French, German and Japanese (see <http://www.wasog.org/education-research/questionnaires.html>).

The minimal important difference (MID) on the SFNSL is 3.5 points for a clinically relevant change over a 6-month period [6].

**Table 2. Small Fiber Neuropathy Screenings List.**

Below are a number of questions about possible complaints. Please circle the answer to each question that is applicable to you. Please give an answer to each question, even if you do not have any complaints at the moment. The aim of this questionnaire is to find out how you experience your complaints. There are no correct or incorrect answers. It is important that you are honest.

*Part 1:* These questions are aimed at finding out *how often* you experience the following complaints.

|  | never | sometimes | variably | often | always |
| --- | --- | --- | --- | --- | --- |
| 1. I have painful arms |  |  |  |  |  |
| 2. I suffer from palpitations |  |  |  |  |  |
| 3. I have problems with my bowel movements |  |  |  |  |  |
| 4. I have difficulties with urinating (either in emptying my bladder or being able to hold my water) |  |  |  |  |  |
| 5. My food does not seem to go down well |  |  |  |  |  |
| 6. I suffer from muscle cramps |  |  |  |  |  |
| 7. My feet and/or hands are colder than I am used to |  |  |  |  |  |
| 8. I have chest pain |  |  |  |  |  |

*Part 2:* These questions are aimed at finding out *how serious* your complaints are.

|  | not at all | slightly | variably | moderately | seriously |
| --- | --- | --- | --- | --- | --- |
| 9. I have the feeling that my food gets stuck in my throat |  |  |  |  |  |
| 10. At night I throw the bedclothes off my legs |  |  |  |  |  |
| 11. I have difficulties with urinating (either emptying  my bladder or being able to hold my water) |  |  |  |  |  |
| 12. I have dry eyes |  |  |  |  |  |
| 13. I have blurred vision |  |  |  |  |  |
| 14. I feel dizzy when I get up |  |  |  |  |  |
| 15. I have sudden hot flushes |  |  |  |  |  |
| 16. My feet and/or hands are colder than I am used to |  |  |  |  |  |
| 17. I have painful arms |  |  |  |  |  |
| 18. The skin of my legs is over-sensitive |  |  |  |  |  |
| 19. I have a tingling sensation in my hands (pins and needles) |  |  |  |  |  |
| 20. I have a tingling sensation in my legs (pins and needles) |  |  |  |  |  |
| 21. I have chest pain |  |  |  |  |  |

**References**

1. De Vries J, Michielsen H, Van Heck GL and Drent M (2004) Measuring fatigue in sarcoidosis: the Fatigue Assessment Scale (FAS). Br J Health Psychol 9 (Pt 3):279-291. doi:10.1348/1359107041557048
2. Drent M, Lower EE and De Vries J (2012) Sarcoidosis-associated fatigue. Eur Respir J 40 (1):255-263. doi:10.1183/09031936.00002512
3. Hendriks C, Drent M, Elfferich M and De Vries J (2018) The Fatigue Assessment Scale: quality and availability in sarcoidosis and other diseases. Curr Opin Pulm Med 24 (5):495-503. doi:10.1097/MCP.0000000000000496
4. Kleijn WPE, De Vries J, Wijnen PAHM, and Drent M (2011) Minimal (clinically) important differences for the Fatigue Assessment Scale in sarcoidosis. Respir Med 105:1388-1395. doi:[10.1016/j.rmed.2011.05.004](https://doi.org/10.1016/j.rmed.2011.05.004)
5. Hoitsma E, De Vries J and Drent M (2011) The small fiber neuropathy screening list: Construction and cross-validation in sarcoidosis. Respir Med 105 (1):95-100. doi:10.1016/j.rmed.2010.09.014
6. Voortman M, Beekman E, Drent M, Hoitsma E, and De Vries J (2018) Determination of the smallest detectable change (SDC) and the minimal important change (MIC) for the Small Fiber Neuropathy Screening List (SFNSL) in sarcoidosis. Sarcoidosis Vasc Diffuse Lung Dis 35;333-341.
